# A Pilot Study of 18F-FLT PET/CT in Pediatric Lymphoma

**DOI:** 10.1155/2016/6045894

**Published:** 2016-05-26

**Authors:** Danny L. Costantini, Reza Vali, Susan McQuattie, Jeffrey Chan, Angela Punnett, Shiela Weitzman, Amer Shammas, Martin Charron

**Affiliations:** ^1^Department of Medical Imaging, University of Toronto, 263 McCaul Street, 4th Floor, Toronto, ON, Canada M5T 1W7; ^2^Department of Diagnostic Imaging, Hospital for Sick Children, 555 University Avenue, Toronto, ON, Canada M5G 1X8; ^3^Department of Pediatrics, Hospital for Sick Children, 555 University Avenue, Toronto, ON, Canada M5G 1X8

## Abstract

We performed an observational pilot study of 18F-FLT PET/CT in pediatric lymphoma. Eight patients with equivocal 18F-FDG PET/CT underwent imaging with 18F-FLT PET/CT. No immediate adverse reactions to 18F-FLT were observed. Compared to 18F-FDG, 18F-FLT uptake was significantly higher in bone marrow and liver (18F-FLT SUV 8.6 ± 0.6 and 5.0 ± 0.3, versus 18F-FDG SUV 1.9 ± 0.1 and 3.4 ± 0.7, resp., *p* < 0.05). In total, 15 lesions were evaluated with average 18F-FDG and 18F-FLT SUVs of 2.6 ± 0.1 and 2.0 ± 0.4, respectively. Nonspecific uptake in reactive lymph nodes and thymus was observed. Future studies to assess the clinical utility of 18F-FLT PET/CT in pediatric lymphoma are planned.

## 1. Introduction

18F-FLT (3′-deoxy-3′-[fluorine-18]-fluorothymidine) is a thymidine analog with uptake that reflects cellular proliferation through the activity of thymidine kinase-1 (TK1), an enzyme that is highly expressed during the synthesis phase of the cell-cycle [1–3]. TK1 phosphorylates 18F-FLT to form negatively charged 18F-FLT-monophosphates which are impermeable to the cell membrane. Since most tumor cells have higher TK1 activity than normal cells, the intracellular trapping of 18F-FLT and accumulation of radioactivity occur [1].

The published literature related to the use of 18F-FLT PET/CT in the pediatric population is limited and restricted to studies in pediatric patients with primary brain tumors [4–8]. We therefore sought to evaluate the feasibility of 18F-FLT PET/CT in an observational study in a small cohort of pediatric lymphoma patients. Our goals were to assess the normal tissue distribution of 18F-FLT and to provide standardized uptake values (SUVs) of lesions demonstrating equivocal uptake on 18F-FDG PET/CT.

## 2. Methods

### 2.1. Study Population

This study was approved by our institution's research ethics board (REB number 1000021766). Enrollment was limited to pediatric lymphoma patients with equivocal 18F-FDG PET/CT findings suspicious for malignancy (see “PET/CT Analysis” below for definition of equivocal). Patients/primary caregivers provided written informed consent. 18F-FLT PET/CT findings were not used to influence clinical management.

### 2.2. Image Acquisition

18F-FDG PET/CT was performed as previously described [9]. Subsequent 18F-FLT PET/CT was performed within 1 to 3 days. The administered 18F-FLT dose (5.2 MBq/kg [0.14 mCi/kg], maximum of 370 MBq [10 mCi] with an accepted 10–20% variation) and scanning protocol were the same as those for 18F-FDG PET/CT. Based on recommended doses in a 55.5 kg adolescent, the estimated effective dose from the additional 18F-FLT PET/CT is approximately 4.3 mSv (0.43 rem) [10].

### 2.3. PET/CT Analysis

PET/CT was analyzed by two licensed nuclear medicine physicians. Regions of interest (ROIs) were drawn encircling the lesion-of-interest on attenuated-corrected PET/CT images [9]. For normal tissue distribution, ROIs were drawn around each organ-of-interest to obtain the maximum SUV. Although no clear SUV threshold has been established for 18F-FDG PET/CT for distinguishing benign from malignant uptake, cutoffs in the range of 2.0–3.5 have been used with high sensitivity and specificity [11–14]. We therefore defined “equivocal” as any area of mildly increased 18F-FDG uptake (Deauville score 3 or 4 [15]) with an SUV ≥ 2.0 but < 3.5, which could not be characterized by normal physiologic uptake, or factors known to cause false-positive uptake (e.g., infection/inflammation, brown fat, or thymic rebound) [14]. 18F-FLT PET/CT was similarly visually inspected for any hyperproliferative lesion(s), taking into account the normal physiologic uptake of 18F-FLT that has been described in the adult population [1, 16].

### 2.4. Standard of Reference

PET/CT image findings were compared prospectively in relation to pathology (when tissue sampling was performed within 1 month of 18F-FDG PET/CT), additional cross-sectional imaging, and/or clinical follow-up.

### 2.5. Statistics

Data are expressed as the mean ± standard error of the mean. Significance was calculated according to Student's *t*-test; *p* < 0.05 was considered significant.

## 3. Results

**Figure 1:**
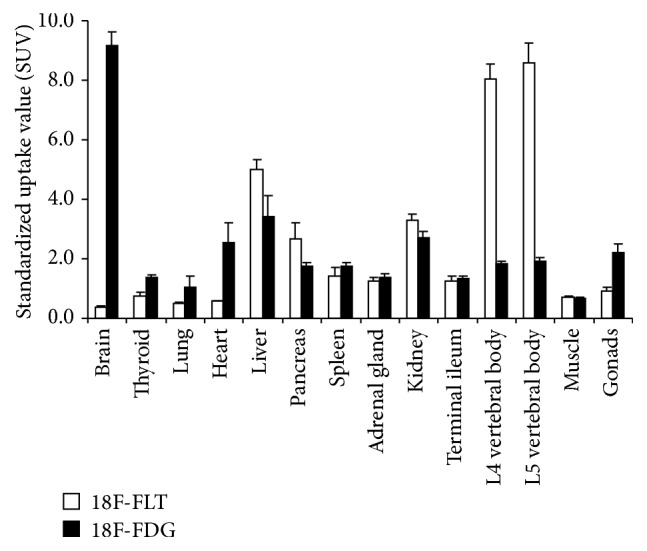
Normal tissue distribution of 18F-FDG (black bars) and 18F-FLT (white bars). *y*-axis is measured in standardized uptake value (SUV).

Between July 2011 and June 2014, twelve patients met enrollment criteria. Consent was obtained in eight patients (5 males and 3 females; median age 16.5 years) who subsequently underwent 18F-FLT PET/CT (Table 1). All patients tolerated the imaging procedure well. No immediate adverse reactions were observed.  Figure 1 shows the normal tissue distribution of 18F-FDG and 18F-FLT. The highest radiotracer uptake for 18F-FLT was found in bone marrow (using L4/L5 vertebral bodies as surrogate tissues) and liver which was significantly greater compared to 18F-FDG (18F-FLT SUV 8.6 ± 0.6 and 5.0 ± 0.3, versus 18F-FDG SUV 1.9 ± 0.1 and 3.4 ± 0.7, resp., *p* < 0.05). Conversely, 18F-FLT uptake in brain, heart, and gonads was significantly lower compared to 18F-FDG (18F-FLT SUV 0.4 ± 0.1, 0.6 ± 0.03 and 0.9 ± 0.1, versus 18F-FDG SUV 9.2 ± 0.4, 2.5 ± 0.7 and 2.2 ± 0.3, resp., *p* < 0.05) (Figure 1).

In total, 15 lesions demonstrating equivocal focal uptake (measuring 1-2 cm) on 18F-FDG PET/CT were subsequently evaluated with 18F-FLT PET/CT (Table 1). The average SUV for 18F-FDG and 18F-FLT for all lesions was 2.6 ± 0.1 versus 2.0 ± 0.4, respectively.

**Figure 2:**
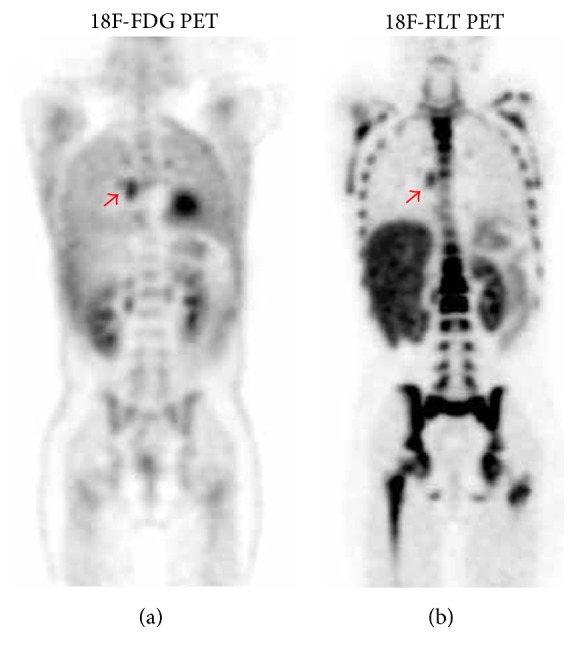
11-year-old female with stage IVA Hodgkin lymphoma (patient 1) demonstrating increased uptake in a right subcarinal lymph node. Biopsy revealed atypical lymphoid hyperplasia.

In patients 1 and 2, 18F-FLT uptake by the index lesion was higher compared to 18F-FDG. Both patients completed chemotherapy at the time of 18F-FLT PET/CT. Biopsy of these lesions demonstrated “atypical lymphoid hyperplasia” (patient 1; see Figure 2) and “normal thymic tissue” (patient 2).

**Figure 3:**
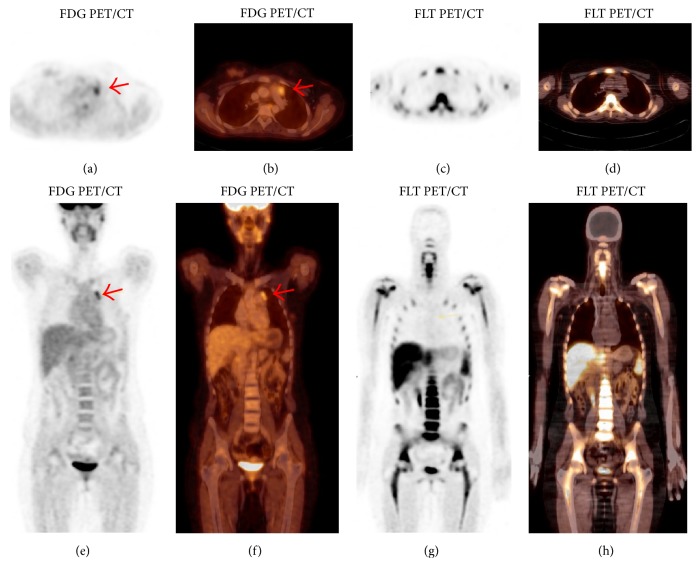
18-year-old female with stage II Hodgkin lymphoma (patient 4) demonstrating increased uptake in a left prevascular lymph node on FDG PET/CT (a, b and e, f) with 18F-FDG SUV 3.0 and no uptake on 18F-FLT/CT (c, d and g, h). Follow-up 18F-FDG PET/CT performed three (3) months later (not shown) again showed evidence of increased metabolic activity in the prevascular region in the upper mediastinum with SUV of 3.1 and not significantly changed compared to the previous study, and no other focus of increased 18F-FDG uptake to suggest disease progression.

In patients 3, 4, and 5, no 18F-FLT uptake in the index lesion was observed and no SUV was calculated (Figure 3). Patients 3 and 4 were mid-treatment at the time of 18F-FLT PET/CT. The index lesion demonstrated negligible or stable uptake on a subsequent 3-month follow-up 18F-FDG PET/CT (data not shown). Patient 5 completed chemotherapy at the time of 18F-FLT PET/CT. A follow-up CT demonstrated an interval decrease in the size of the index lesion.

18F-FLT uptake by the index lesions observed in patients 6, 7, and 8 was predominantly lower compared to 18F-FDG. Patients 6 and 8 completed chemotherapy at the time of 18F-FLT PET/CT, whereas patient 7 was mid treatment. Minimal or no 18F-FDG uptake was observed within any of these index lesions on a 3-month follow-up 18F-FDG PET/CT (data not shown).

## 4. Discussion

To our knowledge, the normal tissue distribution of 18F-FLT in pediatric patients has not been described. Overall, our data reflects that seen in the adults [17], with decreased 18F-FLT uptake in brain and myocardium and increased uptake in liver and bone marrow relative to 18F-FDG [18]. Increased 18F-FLT uptake in reactive lymph nodes was also seen mimicking lymphoma recurrence (patient 1). Troost et al. [19] similarly observed elevated 18F-FLT uptake in head-and-neck cancer patients due to lymphoid cell proliferation within the germinal centers of reactive lymph nodes. Thymus is another lymphoid organ which similarly caused false-positive mediastinal 18F-FLT uptake. 18F-FDG uptake in the thymus secondary to postchemotherapy hyperplasia has been well described in pediatric lymphoma PET/CT [14]; however, 18F-FLT thymic uptake is not as well recognized. Awareness of tumor mimics such as those described, as well as knowledge of the normal tissue distribution of 18F-FLT, is critical in the accurate interpretation of 18F-FLT PET/CT.

We found 18F-FLT PET/CT to be useful in equivocal cases of 18F-FDG PET/CT when little or no perceptible 18F-FLT uptake was seen. Patient 5, for example, was posttherapy at the time of 18F-FLT PET/CT and demonstrated no evidence of recurrent disease on follow-up imaging suggesting that the 18F-FLT PET/CT result represented a true negative finding. Similar findings were obtained in patients 3 and 4; however, the interpretation is confounded by the fact that these patients were mid treatment at the time of 18F-FLT PET/CT. As such the index lesion in these patients could have represented a benign self-limited process versus a malignant lesion with poor FLT avidity and interval treatment response on follow-up imaging. The value of a positive 18F-FLT PET/CT is unknown since no true positive results were observed. This likely reflects the limited number of patients that were examined and the low likelihood of malignant disease being present in equivocal lesions with relatively low 18F-FDG metabolic activity.

Several studies have attempted to define an optimal 18F-FLT SUV for which to separate benign from malignant lesions. Buck et al. [20], for example, concluded that a 18F-FLT SUV cutoff of 3.0 could accurately discriminate between indolent and aggressive lymphomas in adults. If a similar 18F-FLT SUV cutoff is applied in retrospect, it would suggest that the majority of the lesions observed were nonmalignant processes versus (at most) low-grade disease. The lesion in patient 8 had an 18F-FLT SUV > 3.0 and demonstrated complete resolution on follow-up imaging, thus also likely representing a benign etiology. This suggests that a cutoff of 3.0 may be too low of a threshold in our patient series. Indeed, others have suggested higher 18F-FLT SUV cutoffs, for example, in the study by Schöder et al. [21] who demonstrated good sensitivity and specificity (81% and 71%, resp.) for distinguishing indolent versus aggressive lymphoma using a 18F-FLT SUV cutoff > 10.

In conclusion, 18F-FLT PET/CT is well tolerated in pediatric lymphoma patients. 18F-FLT uptake is the highest in liver and bone marrow, whereas minimal uptake in brain and myocardium is seen. Nonspecific uptake can be seen in thymus and reactive lymphadenopathy. Further investigation with a larger number of cases is planned in order to establish meaningful 18F-FLT SUV cutoffs, particularly in the evaluation of pediatric lymphoma.

## Figures and Tables

**Table 1:** Patient characteristics as well as index lesion location, tracer uptake, and reference standard outcome.

Patient	Age, gender	Diagnosis, stage^‡^	Index lesion(s) location	18F-FDG SUV	18F-FDG liver SUV	DS	18F-FLT SUV	Reference standard and outcome^**∗**^
1	11, F	HL, IVA	Right subcarinal LN	2.0	1.2	4	3.3	Biopsy, atypical lymphoid hyperplasia
			Retrocaval LN	2.3		4	4.1	

2	17, M	HL, IVB	Anterior mediastinal mass	2.2	2.6	3	5.0	Biopsy, thymic tissue

3^†^	16, F	HL, IIA	Retroauricular LN	2.5	2.5	3	Nil	Imaging, resolution on follow-up 3 mo 18F-FDG PET/CT

4^†^	18, F	HL, IVA	Prevascular LN	3.0	2.4	4	Nil	Imaging, resolution on follow-up 3 mo 18F-FDG PET/CT
			Prevascular LN	3.0		4	Nil	

5	17, M	HL, IVA	Lung RUL nodule	2.2	2.3	3	Nil	Imaging, interval decrease in size on 3 mo follow-up chest CT

6	14, M	HL, IIA	Jugulodigastric LN	2.7	2.6	3	2.2	Imaging, resolution on follow-up 3 mo 18F-FDG PET/CT
			Jugulodigastric LN	2.4		3	2.7	
			Anterior mediastinal mass	2.6		3	1.7	

7^†^	15, M	HL, IIV	Posterior cervical LN	2.5	2.4	3	1.7	Imaging, resolution on follow-up 3 mo 18F-FDG PET/CT
			Hilar LN	2.5		3	1.9	
			Hilar LN	3.4		4	1.4	

8	17, M	DLBCL, I	Jugulodigastric LN	2.9	2.4	4	2.8	Imaging: interval decrease of 18F-FDG uptake on follow-up 3 mo 18F-FDG PET/CT (SUV 1.5)
			Submandibular LN	2.1		3	3.4	

^*∗*^Histopathology based on biopsy, when available, or follow-up imaging (i.e., 3-month PET/CT or CT scan) was used as reference standards. HL: Hodgkin lymphoma, DLBCL: diffuse large B-cell lymphoma, SUV: standardized uptake value, LN: lymph node, RUL: right upper lobe, mo: month, and nil: no tracer uptake detected. DS: Deauville score, based on the uptake of 18F-FDG within the index lesion using liver uptake for reference. ^†^Patients who were mid chemotherapy at the time of 18F-FLT PET/CT imaging. ^‡^All patients with HL were initially diagnosed pathologically with the nodular sclerosing subtype.

## Abbreviations

18F-FDG: 2-[Fluorine-18]-fluoro-2-deoxy-D-glucose
18F-FLT: 3′-Deoxy-3′-[fluorine-18]-fluorothymidine
PET/CT: Positron emission tomography/computer tomography
SUV: Standardized uptake value
TK1: Thymidine kinase-1.
